# Touching and being touched: where knowing and feeling meet

**DOI:** 10.3389/fpsyg.2023.1097402

**Published:** 2023-07-10

**Authors:** Lawrence Fischman

**Affiliations:** ^1^Department of Psychiatry, School of Medicine, Tufts University, Boston, MA, United States; ^2^Fluence, Woodstock, NY, United States

**Keywords:** transitional states, absorption, minimal and narrative self, touch, depersonalization, psychedelic-induced ego dissolution, mind-wandering, empathy

## Abstract

Philosophers maintain that touch confers a sense of reality or grounding to perceptual experience. In touching oneself, one is simultaneously both subject and object of touch, a template for experiencing oneself as subject and object of intentions, feelings, and motivations, or intersubjectivity. Here, I explore a form of self-touch carefully documented by Winnicott in observing how the infant engages the transitional object. I compare the processes of self-loss in transitional states, including absorption in art, empathic immersion, drug-induced ego dissolution, and depersonalization. I use examples drawn from Rodin, Dante, and the Beatles; research correlating neurophysiological findings with aspects of self-representation; predictive processing-based models; Hohwy’s concepts of minimal and narrative self; Clark’s notion of the extended mind; and phenomenological perspectives on touch, to postulate a role for self-touch in the pre-reflective sense of mine-ness, or grounding, in transitional states.

## Introduction

We spend a great deal of time in states of unguided thought loosely termed mind-wandering. In this subliminal space, we revisit old scenarios and imagine new ones. For such activity to have adaptive or evolutionary value (Corballis, 2013; Smallwood and Andrews-Hanna, 2013), we must simultaneously feel engrossed in it, yet not lose connection altogether with the here and now. How do we readily toggle (transition) back and forth between these disparate worlds?

Winnicott’s acute observations of the infant and intricate knowledge of the adult mind led him to his seminal concept of “transitional phenomena.” His minutely detailed description of the origins of the transitional object suggests that this toggling may have a tactile template.

In the case of some infants the thumb is placed in the mouth while fingers are made to caress the face by pronation and supination movements of the forearm. The mouth is then active in relation to the thumb, but not in relation to the fingers. The fingers caressing the upper lip, or some other part, may be or may become more important than the thumb engaging the mouth. Moreover, this caressing activity may be found alone, without the more direct thumb-mouth union.

In common experience one of the following occurs, complicating an auto-erotic experience such as thumb-sucking:

with the other hand the baby takes an external object, say a part of a sheet or blanket, into the mouth along with the fingers; or

somehow or other the bit of cloth is held and sucked, or not actually sucked; the objects used naturally include napkins and (later) handkerchiefs, and this depends on what is readily and reliably available; or

the baby starts from early months to pluck wool and to collect it and to use it for the caressing part of the activity; less commonly, the wool is swallowed, even causing trouble; or

mouthing occurs, accompanied by sounds of ‘mum-mum,’ babbling, anal noises, the first musical notes, and so on.

One may suppose that thinking, or fantasying, gets linked up with these functional experiences.

All these things I am calling *transitional phenomena*. Also, out of all this (if we study any one infant) there may emerge some thing or some phenomenon – perhaps a bundle of wool or the corner of a blanket or eiderdown, or a word or tune, or a mannerism – that becomes vitally important to the infant for use at the time of going to sleep, and is a defense against anxiety, especially anxiety of depressive type. Perhaps some soft object or other type of object has been found and used by the infant, and this then becomes what I am calling a *transitional object* (Winnicott, 1971a, pp. 3–4).

Though his eventual emphasis in this well-known paper conforms to the general tendency in psychoanalysis to avoid using and writing about physical touch in favor of discussing its meaning and symbolization (Orbach, 2003a,b), Winnicott notably used physical touch and holding in his practice. Here, he clearly grounds his and the reader’s “thinking, or fantasying” in tactile experiences: the minutely detailed description of the movement of the fingers in relation to the lip, independent of the action of the mouth sucking the thumb; the attribution of importance to these caressing finger movements (he uses the word “caressing” four times); their uses; their association with the experience of texture, with ‘taking in,’ with sounds, music, verbalizations, with the body from mouth to anus, and most importantly, with “thinking, or fantasying” (see also Winnicott, 1989).

In physical contact with others as in self-touch, we are both subject and object of tactile sensation (Merleau-Ponty, 1962; Husserl, 1989). In this way, *touching and being touched parallel the process of knowing and being known*, and as I will argue, inform the latter process. Winnicott’s observation of the infant’s apposition of fingers and mouth in the transitional state foreshadows the role of self-touching in navigating subliminal states of consciousness in general. This paper explores *the role of touching in knowing*, or the grounding of experience while losing oneself in the narratives of others, daydreams, the arts, depersonalization, and to a lesser extent in psychosis and psychedelic states. I will argue that self-touch helps us negotiate the transitional space between subjective reality and that which is objectively perceived.

### Rodin

**Figure 1:**
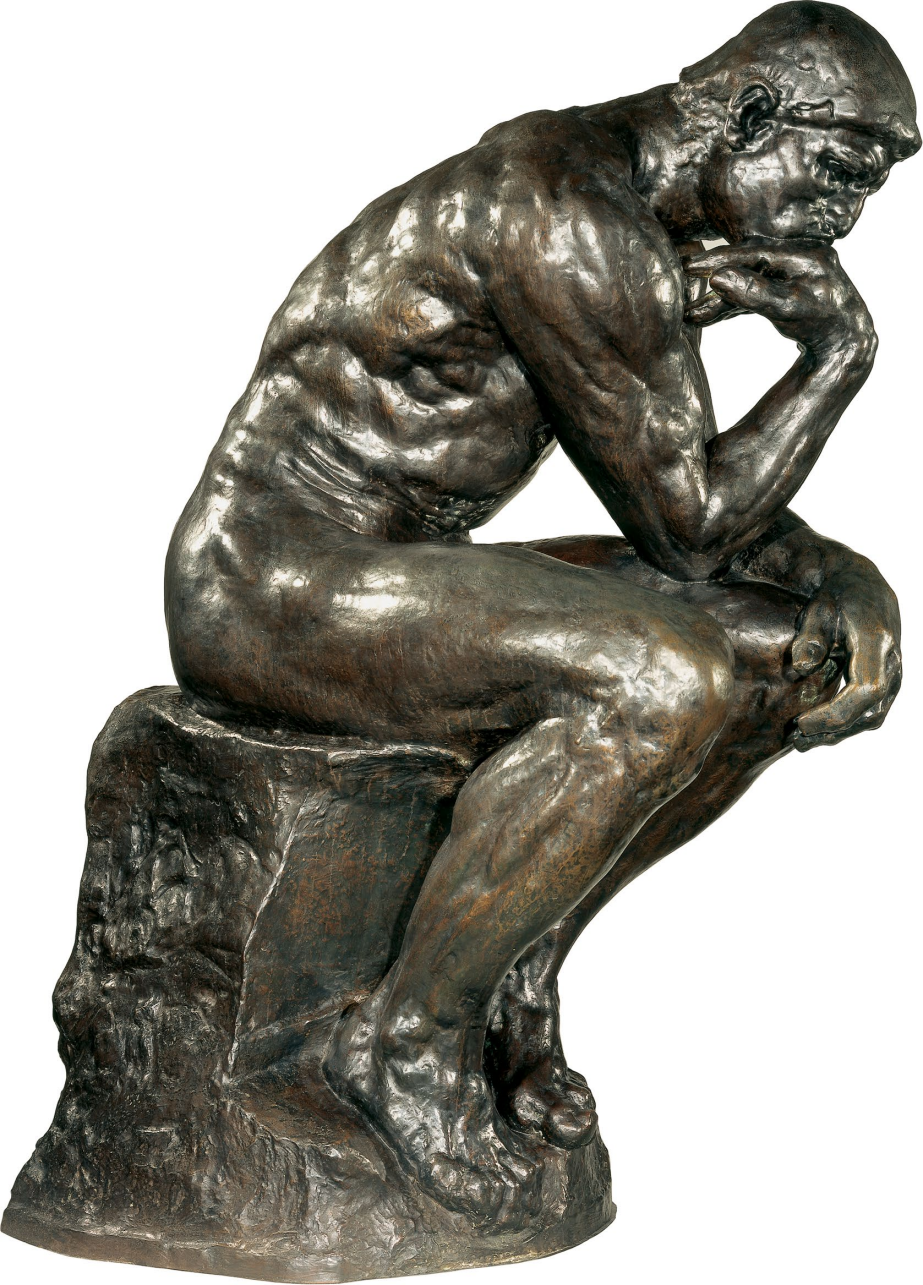
The Thinker, by Auguste Rodin (French, 1840‑1917) Original model 1880; this cast 1904‑1917 Bronze 79 x 37 3/4 x 59 in. (200.7 x 95.9 x 149.9 cm.) Photo credit: The Baltimore Museum of Art: The Jacob Epstein Collection, BMA 1930.25.1. Reproduced with permission.

Why did Winnicott associate this image of the infant with the birth of transitional phenomena? Why do adults often adopt certain positions when “thinking, or fantasying?” What comes to mind is Rodin’s famous sculpture, *Le Penseur* (*The Thinker*), who sits with head resting on hand, knuckles mashed into the partition between its lips (Figure 1).

Rodin originally called his figure *Le Poète* (*The Poet*). It was part of a larger work entitled *La Porte de l’Enfer*, (*The Gates of Hell*). In 1880, Rodin was commissioned to create an entry for a proposed museum for the decorative arts in Paris. His inspiration came from Canto 3 of *The Inferno*, where Dante the pilgrim approaches the threshold of Hell, denoted by the words on the gate, “Abandon every hope, who enter here.”^1^ The pilgrim Dante is quite shaken by the suffering souls he witnesses and turns to his guide, the ancient Roman poet Virgil, for an explanation. Virgil cautions Dante that in entering this world, he must leave all expectation behind:

Here all suspicion needs must be abandoned,

All cowardice must needs be here extinct (Canto 3, lines 14–15).^2^

We can discern in Virgil’s words advice similar to the “flight instructions” given to subjects embarking on a psychedelic experience,^3^ or advice which might pertain to someone attempting empathic immersion of any kind.

Without delving further into the poem, it must suffice to say that Dante, the pilgrim, experiences mixed emotions for the suffering souls he encounters outside the gates, “who lived without disgrace and without praise” (Canto 3, line 36) in an eternal transitional space that is neither Heaven nor Hell. His respect for the divine authority of justice he sees enacted there is mixed with pathos for their suffering, so evident in Rodin’s figures and scenes on the *Gates*.

Rodin was to have delivered the completed *Gates* by 1885, but plans for the decorative arts museum were abandoned, leaving Rodin with no deadline and free to adapt his work to his changing conceptions. In an interview in *Le Matin* published in 1890, Rodin is quoted: “For a whole year I lived with Dante, with him alone, drawing the eight circles of his inferno….At the end of this year, I realized that while my drawing rendered my vision of Dante, they had become too remote from reality. So I started all over again, working from nature, with my models.” Rodin spent 37 years working and re-working the 180 figures in *The Gates of Hell*, which he never completed. Many of these figures, including *The Thinker*, *The Three Shades*, and *The Kiss*, were made into free-standing larger than life-sized statues now familiar to the world.

**Figure 2:**
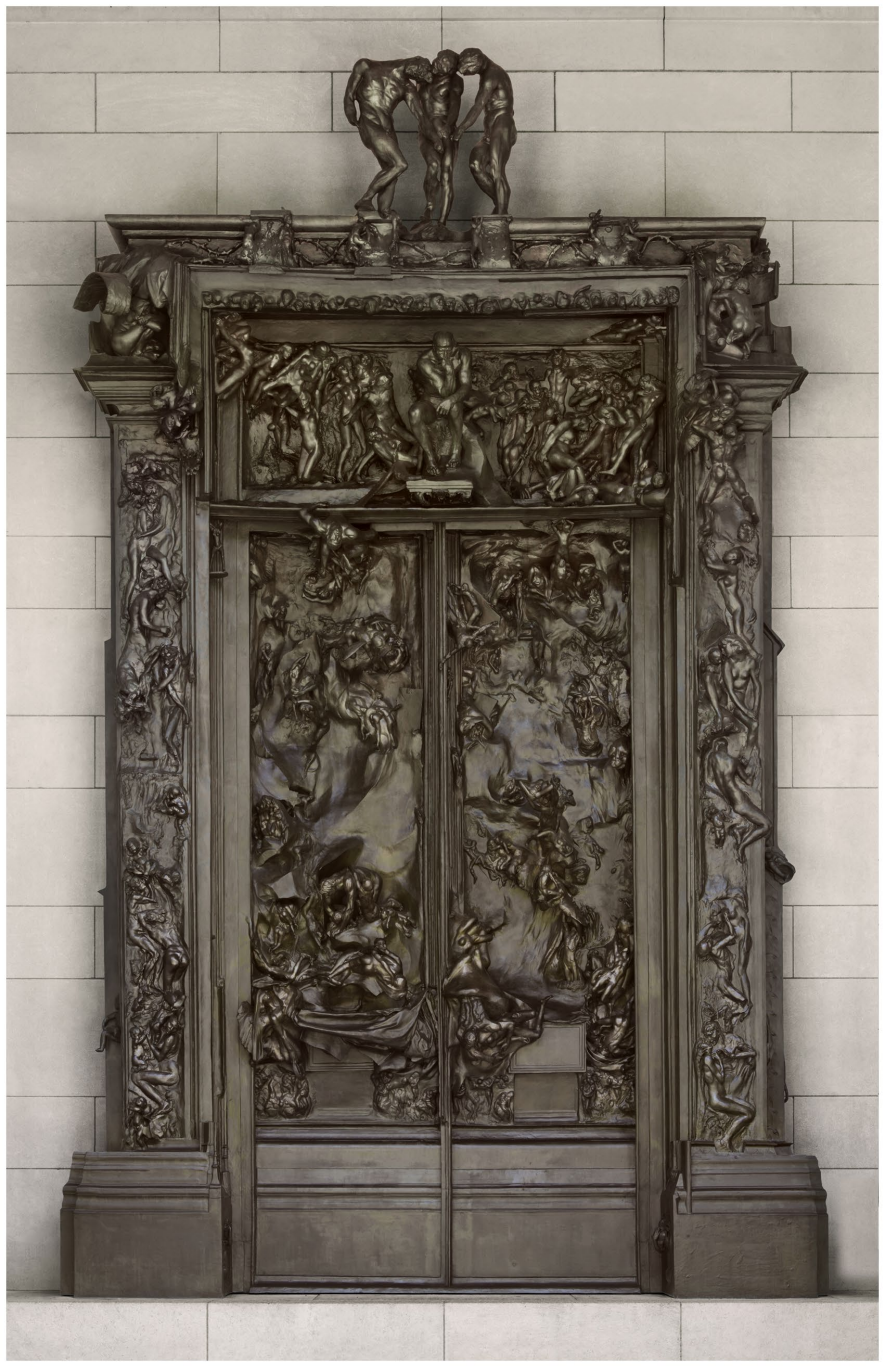
The Gates of Hell, by Auguste Rodin, modeled 1880-1917, cast 1926-1928. Photo credit: Philadelphia Museum of Art: Bequest of Jules E. Mastbaum, 1929, F1929-7-128. Reproduced with permission.

When viewed in its original context, it becomes apparent that the Poet, or Thinker, who is positioned in the center of the sculpture above the *Gates*, is in some relation with the various figures and scenes just behind and around him (Figure 2). The configuration of the sculpture lends itself to an interpretation that these scenes are manifestations of the Poet’s mind, or at least scenes which preoccupy him. In *The Inferno*, a shade’s voice warns Dante the pilgrim of the woes he is about to witness. Rodin transforms these three lines of poetry into *The Three Shades*, whose arms guide the viewer’s gaze toward the Poet/Thinker just beneath them at the top of the portal. It is an entry into a world which absorbed Rodin for the remainder of his life.

### Rodin and Dante

Why did Rodin spend a year of his life with Dante alone? What is he telling us about his internal process in describing his drawing as rendering his vision of Dante, but being too remote from reality? Rodin’s comments suggest that not only was he wholly absorbed by Dante’s poem, but that he became captivated by the process of absorption itself, and how in that process he lost touch with a dimension of reality that may best be described as “grounding.”

Winnicott’s carefully chosen words cited above describe the tactile dimension of the way the infant comes to know something. This system for knowing, assimilated through the fingers and mouth, is rooted in the tactile relationship between infant and mother. Self-touch, the feeling of skin against skin, evokes this developmental template of coming to know. Rodin’s experience with Dante suggests that the absorptive process by which we come to know something is enhanced, may even require, being able to feel something in a tactile, kinesthetic, or embodied way.

One of the two scenes in *The Gates of Hell* which absorbed Rodin most (Elsen, 2003), which has been the subject of so much art, music, and critical discussion of *The Inferno*, is the story of Francesca and Paolo, whom Dante the pilgrim encounters in Canto 5 in the second circle of Hell. Their story, which was well-known in Dante’s fourteenth century Italy, where it took place, is briefly as follows:

Francesca was the daughter of the lord of Ravenna, a city-state at war with Rimini, a rival power in northern Italy. To secure peace, Francesca’s father entered into an alliance with Rimini’s leaders, agreeing to marry his daughter to their heir apparent, Giovanni. The union was an unhappy one: Francesca fell in love with her husband’s younger brother, Paolo, and when Giovanni discovered their affair, he killed them both in a rage (Heil, 2021).

Francesca was tricked into marrying Giovanni, who was said to be deformed or crippled, and in some versions of the tale, ill-tempered, by thinking she was marrying his handsome brother, Paolo, who stood in for him prior to the consummation of the marriage. In *The Inferno*, Canto 5, Dante the pilgrim sees the joined spirits of the lovers swirling lightly in the wind and asks Virgil if he might to speak to them. Francesca, moved by Dante’s interest, tells him their story, including the moment when, while reading a tale of two other adulterous lovers, Lancelot and Guinivere, she and Paolo gazed into each other’s eyes and Paolo kissed her on the lips.

Dante, the poet, invented this detail about the inspiration for the kiss, perhaps to illustrate for the pilgrim the emotional power of narrative and the recklessness of subjugating reason to passion. Its inclusion illustrates the recursive relation between subject and object in Dante’s poem that intrigued Rodin. Dante, the poet, invokes a theme of illicit surrender in the story of Lancelot and Guinivere, which triggers a similar surrender in Francesca and Paolo. In turn, the story of Francesca and Paolo is so powerful that it causes Dante, the pilgrim, to swoon.

After hearing Francesca’s tale, Dante the pilgrim bows his head in deep thought, punctuated by Virgil, in true psychoanalytic mode, asking, “What thinkest?” Here, we have the pilgrim poised as “*Thinker*,” processing Francesca’s story. He has been pondering the irony in how the lovers’ sweet thoughts led to their present suffering. Much literary criticism of this portion of *The Inferno* centers on the themes of lust vs. love; on Francesca’s portrayal of herself as compelled by love rather than taking responsibility for her decision to act adulterously; and on the pilgrim’s possibly misplaced pity for the transgressors. I focus here on the poem as a creative treatment of the process of knowing. The recursive relations between subject and object (which also imply the relation between poet and reader) afford a means by which Dante conveys the complex processes of encountering and taking perspective, of feeling and thinking, of “coming to know.”

### Moments of meeting

After the pause punctuated by Virgil’s question, the pilgrim asks Francesca’s shade how she and Paolo came to know each other’s love. Francesca tells the pilgrim how her gaze met Paolo’s, and how this changed everything. The psychoanalytic concept of a moment of meeting refers to a coming together of two subjectivities that transforms the nature of the intersubjective relationship between the two. It is derived from pre-reflective, implicit relational knowing, the “how to be” with someone, illustrated here in the glances exchanged between Francesca and Paolo. Moments of meeting lead to a new level of knowing and being known (Lyons-Ruth et al., 1998; Stern et al., 1998). Dante’s insertion of Galehaut’s tale creates a narrative of recursive moments of meeting, one serving as the touchstone, as it were, for the next.

That the subject of Dante’s moment of meeting is also a physical coming together, a kiss, adds to the rich resonances that abound between the story, the artists it intrigued, and our investigation of their process. Below, I speculate further about what drew Dante, Rodin and many other artists to the story of Francesca and Paolo, but here I focus upon how the process of “coming to know” relates to Winnicott’s infant.

Winnicott maintains that infant is engaged in “thinking, or fantasying” about her first not-me possession. She uses her sensation of touch just where its end organs are most densely concentrated, to apprehend early not-me objects as she apprehended her first not-me object, mother. Touching one’s fingers to one’s mouth as an adult recapitulates one’s earliest ways of knowing and exploring. It may represent an embodied component of Tronick et al.’s (1998) dyadic expansion of consciousness. It symbolizes the moment of meeting (of minds), but it is not purely symbolic because the tactile sensations in the fingers and the mouth deepen the subject’s sense of reality about the story; they “ground” the story in *felt* experience.

As adults, when we press our fingers against our lips, are we not exploring our connection to the object of our thoughts? Are we not taking in, so to speak, its features, its implications, its relevance, its reality for us? As a thought experiment, try to imagine a complex object without using tactile sensation altogether; no finger to lips contact, no finger to finger or tactile surface to tactile surface contact, or kinesthetic (including facial) movement of any kind. How emotionally real does the object of your contemplation then feel? Even as I compose this thought experiment on a laptop, whenever I pause my typing, I find my fingers softly contacting each other as I contemplate my task. While this thought experiment does not go as far as sensory deprivation tanks do, I suspect the results are related to the latter, in which one’s sense of reality is diminished when sensory input, including tactile information, is sharply curtailed.

I do not propose that this gentile tactile movement or pressure adults engage in while “thinking, or fantasying” supplies specific data about the object of contemplation in the same way that the infant’s fingers, lips, and mouth do. I merely suggest that this activity carries over from its initial ontogenetic role in exploration, and plays a conditioned role in processing transitional phenomena in general. An apt analogy might be the role that unconscious imitation of the target’s facial expression plays during the experience of empathy (Meltzoff and Moore, 1977; Gallese, 2001; Meltzoff, 2007). One can suppress such imitation and still experience empathy, but perhaps not as effortlessly or fully. In the same way that mirror neurons participate in the empathic process, it is possible that some yet to be characterized glabrous skin equivalent of C tactile (CT) afferents may participate in transmitting information about the imagined experience of the object under contemplation. The tactile activity of fingers and mouth may be neither necessary nor sufficient to process transitional experience within contemplative states, but may facilitate it.

### A day in the life

Stone (2012) named “*A Day in the Life*” the greatest of all Beatles’ songs. The song, co-written by John Lennon and Paul McCartney, illustrates just how permeable is the barrier between everyday reality and fantasy. It does so primarily in its arresting aural transitions and shifts in intensity and cadence, but it also conveys this directly in one of the song’s lyrics: “Somebody spoke and I went into a dream.” The line must be heard in its recorded form to fully appreciate its instantaneous transitional effect. Nevertheless, the idea is that in ordinary waking life we are never more than an utterance away from a dream, or from entering the “potential space” between the subjective object and the object as perceived by others (Winnicott, 1971b). As our boundaries blur in gaining entry into this space, it may help to retain some grounding in everyday reality. That grounding, I suggest, may be tactile.

The thin line between reality and fantasy, even between life and death, is conveyed in the song’s first stanza, with the opening line: “I read the news today, oh boy/About a lucky man who made the grade.” We soon learn that the song’s protagonist, much like Dante, the pilgrim, when he meets the shades, Francesca and Paolo, is trying to find meaning in a story of a life which ends in a sudden, cruel death.

And though the news was rather sad

Well, I just had to laugh

I saw the photograph

He blew his mind out in a car

He didn't notice that the lights had changed

A crowd of people stood and stared

They'd seen his face before

Nobody was really sure if he was from the House of Lords

Lennon traces this part of the song to learning about the death of Tara Browne, a socialite acquaintance (and friend of Paul McCartney) and the heir to the Guinness fortune who was killed in a car crash (Howard, 2016). Lennon was sitting at his piano when he came across an account of Browne’s death in the *Daily Mail*. He promptly incorporated the story into a song. There are many versions of how the accident occurred, ranging from Browne driving at very high speed under the influence of alcohol and other drugs and not noticing a traffic light, to his driving only slightly fast and swerving into a parked car to avoid a speeding car which ran a light at the intersection. There are many versions of the Francesca and Paolo story, too, and that is the point. Both are tragic stories involving well-known, affluent people, which incite the imagination.^4^ They are magnets of absorption for those who sublimate their own conflicts and predicaments by living vicariously through the lives of others, which is to say many of us.

As in Dante’s *Inferno*, there is a relation between the song’s structure and content that illustrates the way we explore ambiguity or discover meaning through vicarious engagement in fantasy. Just as the protagonist of the poem is not Dante the poet, but Dante the pilgrim, an alter-ego who negotiates the world of dream and myth, so the performers of the song are not the Beatles themselves, but Sgt. Pepper’s Lonely Hearts Club Band (the title of the album on which “A Day in the life” appears), an invented Edwardian military band that served as the band’s alter-ego. Paul McCartney describes the imaginative origins of the band’s alter-ego, and the purpose it served:

"I was on a plane with our roadie, and we were eating, and he said, 'Can you pass the salt and pepper?' I thought he said 'Sergeant Pepper,'" McCartney said. "We had a laugh about that. And the more I thought about it, Sergeant Pepper - that's kind of a cool character."

McCartney says the concept of "Sgt. Pepper," the band's eighth studio album, released in 1967, was freedom from expectation.

"I said it'd be great to make an album like we're alter egos of ourselves," he told the producer Rick Rubin, who costars in the docuseries. "So we don't have to think, 'This is the Beatles making an album.' There's no pressure of, 'What do the Beatles need to do now?' This is just some other band" (Ahlgrim, 2021).

McCartney elsewhere elaborated on the circumstances which may have prompted the use of an alter-ego:

When the band concluded touring in 1966, the four members took some time apart to pursue other passions. McCartney went traveling alone, often outfitted in a mustachioed disguise.

"Nobody recognized me at all. It was good, it was quite liberating for me," he told the biographer Barry Miles for the 1997 book "Many Years From Now." "I was a lonely little poet on the road with my car" (Ahlgrim, 2021).

The creation of alter-egos and disguises free Lennon and McCartney from a sense of expectation, in some sense from their identities as Beatles, and facilitates their creative process. As we will see later, attenuating the self-representation facilitates empathic immersion/absorption. “A Day in the Life” evocatively depicts the creative process itself; its music and lyrics illustrate how readily the boundary between subjective and objective reality may be crossed.

### Self-other overlap

How does tactile sensation, or assuming the bodily position of Rodin’s *The Thinker*, affect the process of “thinking, or fantasying” that takes place when one reads Dante’s *Inferno*, when one encounters the story of Francesca and Paolo, or of Tara Browne’s death? I mentioned earlier Rodin’s statement that he spent a year of his life alone with Dante, but in the process, drifted too far from reality. I underscored the recursive process contained in the relations between Rodin, Dante (the poet), Dante (the pilgrim), Virgil, Francesca, Lancelot and Guinivere, each of whom are absorbed in understanding and relating the experience of another to their own experience. The effort engages the process of empathy, which requires what has been termed, if clumsily, self-other overlap (Preston and Hofelich, 2012).

Most discussions of empathy and intersubjectivity in the philosophical and psychological literature claim that self-other distinction is maintained during the process of *Einfuhlung*, or “feeling into” (Lipps, 1903; Freud, 1921; Kohut, 1959; Husserl, 1973, quoted in Zahavi, 2001; Basch, 1983; Zahavi, 2001; Gallese, 2003, 2014 reviewed by Ganczarek et al., 2018, and Papadopoulou, 2022), but this distinction may be more tenuous than the literature implies. Such tenuousness is implied in the distinction drawn between affective and cognitive components of empathy (Lawrence et al., 2007; Ebisch et al., 2022), where involuntary, or pre-reflexive affective resonance between two individuals may blur the subject-object boundary, which is then affirmed through effortful, cognitive processes. In the following, “empathy” refers to both processes, including some mind-wandering processes which link them.

Bird and Viding (2014) have developed an informational processing model of self-other overlap in empathy. During “emotional contagion” or “affective resonance” between two individuals, which may be mediated by the mirror neuron system, the empathizer receives “psychophysiological cues” alerting him that his feelings are his own. To facilitate empathy, a “self-other switch” must reset from its default position of “self-focused” to “other-focused.” This attentional, motivational shift enables the empathizer, who feels the other’s feelings but does not know they “belong” to the other, to suppress self-related expectancies (needs, wishes, personal feelings, etc.) in order to gain access to the other’s frame of mind. This biases and “tags” his affective response toward what is regarded as appropriate for the other. In this account, the “mine-ness” of affective experience is never in doubt, and volitional effort must be made to attribute the source of one’s feelings to the other.

However, the sense of mine-ness is not always so clear-cut, it is sometimes just a best guess (Seth and Tsakiris, 2018). I suggest that the “psychophysiological cues” which confer a sense of mine-ness to shared feelings are reinforced by self-touch (see below).

There is phenomenological evidence that given deliberately misleading multisensory input, including tactile input, one can mistake another’s hand (Botvinick and Cohen, 1998), or even another’s body for one’s own (Petkova and Ehrsson, 2008). Under the free energy principle (FEP)/predictive coding account (Friston, 2005, 2010), what is taken for self and not-self is determined by the fit between prior expectation and incoming interoceptive and exteroceptive information. How heavily sensory information is weighted depends upon the precision it is accorded, which in turn depends upon whether incoming signals suggest a narrow versus a broad range of prediction possibilities: the narrower the range, the greater the confidence one has in the accuracy of the signal (Tsakiris, 2017). Posterior beliefs about mine-ness are simply a best guess as to “what is most likely to be ‘me’” (Limanowski and Blankenburg, 2015; Tsakiris, 2017).

The reason for this slight digression is that the process of “thinking, or fantasying” often involves intersubjectivity, or shared states of mind. The subject tries to apperceive the contents of another’s mind. The success of this effort requires a voluntarily blurring of the self-other boundary, by attenuating the precision of self-representations. The subject attempts to center his experience in the body and mind of the other. The rubber hand and full body illusions can be viewed as extreme examples of how it is possible to re-imagine the self-other boundary in such a way that another person’s experiences can be perceived as one’s own. While the specialized laboratory circumstances that produce this illusion are unlikely to arise spontaneously, and while it is likely that in ordinary empathy the self-other distinction is retained to some degree (Zahavi and Rochat, 2015), it is still possible to achieve significant, even dream-like absorption in the imagined experience of the other.

One may even argue that true empathy requires a trial stage of full imaginative absorption in the world of the other, generating a model untinged by self-representation, which may explain the occasional sense of uncanniness about intersubjective knowledge attained this way. This dream-like absorption is based on the same dynamics as absorption in play and cultural experience, and its posterior beliefs, while less constrained by prior expectation, and therefore more novel, are subject to the same illusion-disillusionment process as these transitional phenomena, wherein we agree not to ask the empathizer if he or she “created” or “found” the contents of the other’s mind (Winnicott, 1971a; Fischman, 2022).

Individuals are likely to have unique strategies and skills for mediating such absorption. The largely tactile process described by Winnicott at the outset of this paper represents one of the earliest of such strategies. The phrases “have a feeling for,” being “touched by,” and “losing touch” are some of many idioms in English (see Montagu, 1971, pp. 10–13) which denote the phenomenological link between the sense of touch and knowing. Research is beginning to focus on its importance in prenatal and early post-natal shared experience. “Well before we share experiences by ‘meeting’ other people’s minds distally via vision, we literally ‘meet’ them via proximal channels such as intersubjective touch” (Ciaunica, 2017, p. 193). I suggest that the infant’s tactile exploration of the first not-me objects that Winnicott describes as a key component of the origin of transitional phenomena is continued (in some individuals) in the process of empathic absorption, and pre-reflexively enhances the process of “getting a feel” for the object of interest. The reciprocal sensations of touching and being touched bring the subject closer to a sense of intersubjectively knowing the object. It is as if the contemporaneous experience of touching and being touched, reproduced in caressing self-touch, promotes a reassuring sense of familiarity within alterity, allowing deeper immersion within the other’s narrative, and confers a sense of embodiment and therefore “reality” to empathically evoked thoughts and feelings. This is analogous to embodied simulation as described by Gallese (2001, 2009), as though what is being simulated through tactile pressure are inferred aspects of emotional tension in the other.

### Depersonalization

Recent study and speculation about sensory attenuation in depersonalization disorder (DPD) may have some bearing upon the dynamics of this disorder and the possible role of touch in its treatment. It is discussed here because it is essentially a syndrome of losing the sense of being “grounded,” which, I maintain, is afforded or enhanced in some way by self-touch. Its etiology may have implications for the dynamics of self-touch during empathic absorption and other transitional phenomena. Depersonalization offers an intriguing model for study because it may represent an early phase of what takes place in schizophrenia (Jacobson, 1959, p. 583; Parnas and Handest, 2003; Sass and Parnas, 2003), and an early phase of psychedelic-induced ego dissolution. The following statement, taken from Simeon and Abugel (2006) typifies depersonalization: “I do not feel ‘grounded.’ I look at this body and cannot understand why I am within it. I hear myself having conversations and wonder where the voice is coming from. I imagine myself seeing life as if it were played like a film in a cinema” (p. 15).

In depersonalization, one’s sense of “presence,” defined as “the subjective sense of reality of the world and of the self within the world” (Seth et al., 2012), is diminished. The phenomenological consonance or identity between the “I” who experiences, and the physical body in which the “I” resides is lost. This is often described as a split between the observing ego and the participating self, or a split between self-as-subject and self-as-object. It results in feeling as if one is observing oneself from a detached perspective, even from outside or above oneself. This phenomenological “de-centering,” a sense of partial translocation from the body, of experiential distance from the self-representation, links the dynamics of depersonalization with those of self-other overlap.

Numerous accounts attempt to explain this split. In the older psychoanalytic, literature, depersonalization was attributed to a “decathexis” of the ego boundary (Federn, 1952). In general, psychoanalytic accounts emphasize a defensive function of depersonalization (Oberndorf, 1950; Jacobson, 1959; Stamm, 1962; Arlow, 1966), a protection from overwhelming anxiety, associated with traumatic or otherwise unacceptable aspects of self-experience. Current research on depersonalization centers on predictive processing accounts. These have emphasized imprecise interoceptive prediction signals (Seth et al., 2012); a lack of presence related to loss of predicted affect (Gerrans, 2018); a loss of allostatic control (Deane et al., 2020); and a failure to attenuate predicted somatosensory signals during active inference (Ciaunica et al., 2021).

The relation between predictive processing accounts and the phenomenology of depersonalization is given in the following first-person account: “If I quieten my mind, I can still almost taste the color and richness of life as I knew it before that point; the feeling of being your own agent of change, the feeling of plotting a course through life, and the sense of expectation” (Ciaunica and Charlton, 2018). Charlton implies that a central feature of depersonalization is her lack of any sense of expectation. Predictive processing is all about expectation, generating models derived from prior experience to make probabilistic inferences about new and potentially destabilizing situations.

This account holds that in order to resist a natural tendency toward entropy, living organisms have evolved self-organized homeostatic mechanisms which must be maintained within relatively narrow biologically determined bounds in order to insure survival (Clark, 2013). To maintain homeostasis, an organism must sample its environment and infer the likely causes of perturbations from homeostatic set-points. Any discrepancy, or “error” is dealt with by perceptual inference, in which the model is updated to conform with new data, or by active inference, in which the organism performs actions which ensure that new environmental sampling conforms to its model. These complementary strategies may be summarized as changing the model to fit the world, or changing the world to fit the model (Seth, 2013).

In active inference the brain must suppress kinematic information related to its action, i.e., all of the sensory information, including kinesthetic and proprioceptive data, brought about by its movement, such as turning one’s head to view an object from a different angle. The suppression of this information renders the experiencing of the object “transparent,” or direct, unmediated. It is like looking out at the ocean without being aware one is looking through a glass window.

In depersonalization, suppression of kinematic and other somatosensory information is impaired, possibly because of aberrant precision weighting (Ciaunica et al., 2022). The subject loses a sense of transparency regarding the bodily processes involved in active inference. Extending the earlier analogy, the sense of presence one has with respect to the ocean would be diminished by awareness of the glass. In depersonalization the subject is conscious of the somatosensory information generated by turning one’s head. This may lead to exaggerated focus on the embodied self, and eventually to a dissociation of the observing and participating selves. This is similar to the early stages of psychedelic-induced ego dissolution described by Savage (1955) and Klee (1963), in which subjects become conscious of somatic sensations and processes that are normally automatic, ignored.

In addition to undermining transparency or one’s sense of presence, faulty attenuation of somatosensory information during active inference may also compromise one’s sense of agency (Blakemore et al., 1998; Brown et al., 2013), another central phenomenological disturbance in depersonalization as well as psychosis. Agency is a key component of the pre-reflective minimal self, a consciousness of oneself as an immediate subject of experience (Gallagher, 2000; Moore, 2016). In depersonalization, and even more so in psychosis, excessive attention to self resulting from a failure to attenuate somatosensory sensory feedback of intended acts leads to a confused sense of agency (Ciaunica et al., 2021, 2022), as in: ‘I do not normally think about how to reach for a mug; the fact that I now seem to have to think about this makes me feel less in control of my body.’ In psychosis, this may be elaborated into: ‘Sometimes I feel as though someone else must be controlling my movements’ (Fletcher and Frith, 2009). The fear of losing control may be at the root of depersonalization (Ciaunica et al., 2021). Attenuating somatosensory signaling is relinquishing one’s monitory control of one’s body. In this context, one can speculate that self-touch may play a compensatory grounding role during somatosensory attenuation. This may account for why people with depersonalization sometimes slap or pinch themselves (Colombetti and Ratcliffe, 2012) as if to try to regain a sense of presence by awakening tactile sensitivity. Interestingly, a preliminary study suggests that affective self-touch (see below) may enhance the vividness of perceptual experiences and reduce disembodied and unreal feelings in people with depersonalization disorder (Ciaunica et al., 2023, unpublished study).

### From depersonalization to absorption

Colombetti and Ratcliffe (2012) make useful distinctions between the various types of altered bodily feelings which occur in depersonalization, but state that what they all have in common is an altered “existential feeling,” or “how one finds oneself in the world” (p. 147). They quote a first person account in which a subject with depersonalization states, “[f]amiliar things look strange and foreign … They’re just shapes, objects, things, with no personal connection to me” (p. 147; quotation taken from Simeon and Abugel, 2006). Colombetti and Ratcliffe add that this change in how one finds oneself in the world “is inextricable from a change in bodily self-awareness” (p. 147).

Individuals with DPD often reach a distressing but stable state in which one feels disconnected from one’s body, yet not fully estranged from it. There is no question about whose body it is, even though the way it feels “mine” is greatly altered (Hunter et al., 2003; Colombetti and Ratcliffe, 2012; Ciaunica and Charlton, 2018). As mentioned above, depersonalization, or altered presence and transparency, may herald the onset of schizophrenia, in which close phenomenological scrutiny reveals a primary disturbance of “ipseity,” or “the experiential sense of being a vital and self-coinciding subject of experience or first person perspective on the world” (Sass and Parnas, 2003; Henriksen and Parnas, 2017). This is manifest in “hyperreflexivity,” a form of “exaggerated self-consciousness in which a subject or agent experiences itself…as a kind of external object,” or else “diminished self-affection,” or a “diminishment of …. the sense of basic self-presence” (Sass and Parnas, 2003, p. 148). In schizophrenia, this disturbance in ipseity precedes the development of paranoia, hallucinations, and delusions.

Significantly, depersonalization disorder is often triggered by using drugs with hallucinogenic properties (Simeon and Abugel, 2006, p. 15). As mentioned earlier psychedelic-induced ego dissolution progresses through a stage of depersonalization, or exaggerated consciousness of normally automatic (attenuated or suppressed) bodily processes on the way to full ego dissolution (Savage, 1955; Klee, 1963). In depersonalization disorder, it is as if the process of ego dissolution is arrested halfway. At this midway point, agency, while drastically altered in its *felt* quality, is partially preserved. It is as though the individual, experiencing self-other overlap as loss of control, as disintegration of self, defensively resists the process, does not surrender to it. The “hyperreflexivity” (excessive focus on somatic and interoceptive signals) reported in depersonalization may represent a partly successful effort to cling to a self-representation experienced as threatened, perhaps by opposing the reduction in sensory precision weighting necessary to achieve transparency in exploring transitional phenomena (Limanowski and Friston, 2020; Ciaunica et al., 2022) and mind-wandering. Insofar as presence is necessary for meaningful empathy, it is noteworthy that people suffering from depersonalization disorder have limited capacity for empathic immersion (Lawrence et al., 2007).

High dosages of psychedelics may bring about full disintegration of the narrative self (Letheby and Gerrans, 2017; Milliere, 2017). If one tracks the sense of presence through this process, it is altered and often diminished at the midway point, or when the continuity and cohesion of self-representations is threatened or compromised, but it is heightened with full ego dissolution, when things often feel “realer than real.” At this point, perception feels unmediated; “transparency” is greatest; subjects feel a heightened sense of connectivity with their surroundings, fully “in touch,” as it were, as though “all is one.” Thus, one’s sense of presence, the subjective sense of the realness of one’s world (Metzinger, 2003; Seth et al., 2012), is greatest when self-attenuation is virtually complete (Limanowski and Friston, 2020).

Speculatively, empathic immersion or absorption in a narrative or work of art requires somatosensory attenuation—in FEP terms, an underweighting of the precision of sensory evidence of the self-model, or diverting attention from self-generated acts (Brown et al., 2013) —to achieve presence, or a sense of reality in the other’s world. In its extreme, as in psychosis or psychedelic-induced ego, dissolution, self-representation, and with it a sense of agency, is deactivated altogether (Fletcher and Frith, 2009; Adams et al., 2013). Mind-wandering may require a “self-representational blink” (Metzinger, 2003; Limanowski and Friston, 2020) of the type which is extended in these other conditions. Self-touch, especially if steadily applied, grounds one as both subject and object of experience during sensory attenuation, affording a sense of presence and agency while transiently freeing attention for unconstrained by self-representation (self-touch during absorption is nearly always unconscious).

It is tempting to imagine that this is one of the functions of self-touch during states of absorption. In addition to supplying a background of agency, the “grounding” function of self-touch may consist in translating the affective experience of another into felt vital qualities. In early development and throughout life, vitality affects are conveyed cross-modally via contours of activation, timing, and intensity (Stern, 1985). This cross-model transmission, which often includes intensity contours of touch, is the basis for affect attunement, a way of describing the intersubjective emotional resonance which may take place between two individuals. Subtle variations of pressure during self-touch may create a tactile substrate or analog for the dimensional contours of affect encountered during absorption or empathic immersion. This dovetails, with the work of Ruggieri et al. (1986) regarding muscular “decodification” of visually observed emotions of others (see also Ganczarek et al., 2018), and of Clynes (1980), who used a finger pressure-driven machine to transduce the intensity signatures of emotion (see Papadopoulou, 2022).

## Self-awareness in “thinking, or fantasying”

As we saw in the last section, “presence” or one’s sense of reality is inversely related to self- reflection, or self-awareness. What happens to self-awareness when one is “thinking, or fantasying,” absorbed in a complex narrative? Using a predictive processing model similar to Ciaunica et al. above, Hohwy (2007) hypothesizes that the “default state” of the sense of a narrative self^5^ is sustained by being poised between “pondering one’s role in a given task and forgetting oneself in the task” (p. 3). Hohwy cites functional brain imaging studies (Gusnard et al., 2001; Raichle et al., 2001) which show an orthogonal relationship between the default mode network (DMN) and the task positive network (TPN) in parallel with this “seesaw” (p. 11) between self-reflection and selfless immersion in mental tasks. Milliere (2017) states that DMN-TPN orthogonality or “anti-correlation” might be necessary to maintain a clear distinction between what is internal/self-related and what is external/other-related at the personal level. Hohwy’s model involves a “pondering self:” a *Thinker* caught in a default state between self-reflection and self-immersion. This is precisely the position of someone who is engaged in vicarious introspection, or empathy: Dante, the pilgrim, before Francesca and Paolo; Rodin, constructing *The Gates of Hell*, before Dante, the poet, etc. In Hohwy’s terms,

Before one engages actively with a task, attends to its components and loses oneself in it, one must figure out what the task is all about, what one’s role is in this situation. This is a kind of theorising where one needs to arrive at good hypotheses that make sense of the situation relative to one’s own role in it…. The system needs to arrive at some relatively probable generative models for perception and action that explains the data and probabilifies the desired state. The nature of the reflective self… is as a pondering self in search of good high level hypotheses with a high prior probability, that can generate good predictions about what happens in complex situations, and that will be fruitful for bringing about the desired state (p. 11).

The subject uses self-reflection to generate predictive models of what it is like to have the other’s experience; that is, what it is like to be the protagonist in a situation that resembles the other’s situation (Maibom, 2022). When such a prediction results in minimal prediction error, the subject shifts out of self-reflection and loses himself (becomes absorbed) in a narrative based upon the other’s situation, which later serves to update predictions. The salient point here, which contrasts with the pathological process of depersonalization, is that in health, the pondering self willingly activates and de-activates the self-representation as one imaginatively, empathically engages the experience of another. In predictive coding terms, absorption, the willing suspension of expectation, consists in a transient under-weighting of precisions associated with prior expectations. The subject’s sense of presence is retained even while wholly absorbed in an object. This switching corresponds to the activation and deactivation of certain midline brain structures that comprise the default mode network (Gusnard et al., 2001; Raichle et al., 2001; Northoff et al., 2006; Northoff and Panksepp, 2008; Nelson et al., 2009), a brain network consisting mainly of hubs in the posterior cingulate cortex (PCC)/precuneus, medial prefrontal cortex (MPFC), and inferior parietal lobule (IPL), which is active during rest (i.e., mental time-traveling, autobiographical memory, etc.), or what is subsumed in this paper under the heading of mind-wandering (Smallwood and Schooler, 2015), and deactivated in relation to goal-directed thinking.

Imaging studies have shown that mindfulness training can affect the ability to switch back and forth between midline structures associated with narrative self and those associated with the immediate agentic ‘I,’ i.e., the structures associated with the “split” between the functional experiences of self by subjects during depersonalization (Farb et al., 2007). Functional imaging studies monitoring engagement in the practice of loving kindness meditation, a form of “selflessness,” can distinguish novice from experienced practitioners (Garrison et al., 2014). If switching into selflessness can be learned or trained, so it may be unlearned, or go awry (Britton, 2019; Deane et al., 2020). Brewer et al. (2013) have analyzed functional imaging evidence which suggests that the posterior cingulate cortex (PCC), a hub in the default mode network, is activated while “getting caught up in experience,” that is, involuntarily engaging in self-reference while attending to other-related tasks or narratives. These tasks or narratives include social evaluation, evaluating moral dilemmas, benevolence, compassion, and the desire to help others in need, or narratives related to justice, such as fairness, impartiality, and the desire to liberate others from injustice” (p. 3). These could serve as a menu of mental tasks for Dante the pilgrim as he engages Francesca’s shade in *The Inferno*. Conversely, the PCC is de-activated when not caught up in experience, i.e., when engaged in present-centered awareness or meditation. Ulrich et al. (2014) find that flow experiences (absorption without self-reference) are associated with decreased medial prefrontal cortex (mPFC) activity, which is tightly coupled with the PCC (Davey et al., 2016). Uncoupling between these two areas is posited to reduce self-referential thinking in psilocybin-induced ego dissolution (Carhart-Harris et al., 2012).

But in ordinary states of consciousness, disengaging self-reflection from the evaluation of experience is impossible to sustain. Hohwy states, “Without a sense of who one is in terms of one’s plans and preferences there would be no sense of a reflective self as we know it, but it is also difficult to imagine our sense of a cohesive self without it being put to use on a set of tasks to selectively engage ourselves in, and lose ourselves in” (p. 11). This position brings us back to Rodin spending a year alone with Dante, and in effect, losing himself in the process. Were Hohwy a sculptor, he might have seated his *Thinker* on a seesaw.

Hohwy suggests that the narrative or “reflective” self alternates between dominant and negligible roles in predictive processing precision weighting. Though it may be premature to speculate exactly what aspects of predictive processing go awry during depersonalization [Seth et al., 2012 posit “imprecise interoceptive predictions”], there may be heuristic value in construing its relatively unchanging “midway” position on the path to ego dissolution as a kind of “stuck-ness,” a lack of fluidity in transitioning between orthogonally related states (in EEG studies) of self-reflection and self-immersion (Ciaunica and Safron, 2022). It is precisely this fluidity that may underwrite the mind’s capacity to wander: to enter into and leave transitional states, or engage in empathic immersion in the experience of another. In this vein, individuals with DPD show deficits in adopting another’s point of view and feeling compassion and concern for others in complex scenarios, which may be related to deficits in interoceptive awareness (Sedeño et al., 2014).

### A psychodynamic view of pondering and depersonalization

What if the process of relinquishing the self-representation has psychodynamic underpinnings? If engaging in empathic immersion requires a voluntary “blurring” of self-other boundaries, or, following Hohwy (2007), a seesawing between “pondering one’s role in a given task and forgetting oneself in the task,” is it possible that surrendering the psychodynamically-defended self-representation evokes a dread of the unknown, a fear of what lurks within, which may be resisted? Ciaunica et al. (2022) propose that

high levels of uncertainty and unpredictability may result in feelings of “losing control” over one’s bodily self and actions, triggering compensatory sub-optimal mechanisms of over-control of one’s self and bodily actions. Paradoxically however, as we saw earlier, sense of agency crucially depends on the ability to leave the self in the background (i.e., sensory attenuation).

In this model, clinging to a sense of control during initial phases of ego dissolution may defeat the process of self-surrender necessary to attain empathic immersion and “self-other sharing.” This may be caused by avoidance sensitivity during the process of ego dissolution, as Wolff et al. (2020) have described during psychedelic-induced ego dissolution. Attempted avoidance during ego dissolution seems to promote greater association to related aversive contents, i.e., an intensification of that which is feared. Wolff et al. (2022) suggest, “The attempt to suppress an arising emotion may induce unsettling bodily sensations, sinister imagery, and so on. Conversely, shifting from avoidant responding toward more acceptance can lead to immediate relief from such distress.” Such a shift or surrender may be prevented by fear or self-loathing. As Blewett (1970, p. 347) writes (in relation to LSD in group therapy),

Because of the psychological proximity of the self–concept and its defenses, this active surrender calls for undergoing and overcoming the ultimate fear that is locked in each man's heart: If I should come to know myself completely and still hate and revile myself – what then? What if the self is unacceptable, completely unwanted – an entity without purpose or meaning?

The deeper and more anguished my self-hatred, the more I am likely to fear the ultimate revelation of myself to my own scrutiny.

Only with a complete de-activation of the self-representation does the vicious cycle of avoidance and aversive associations stop. But in depersonalization, that stage is not reached.

Gerrans (2018) endorses an affective model of depersonalization which integrates the predictive processing model with Billon’s (2018) view of “mine-ness,” or the feeling that one’s experiences belong to oneself. Gerrans’s account is not a psychodynamic one, but lends itself to a psychodynamic interpretation. In Gerrans’s view,

DPD arises when the world unpredictably and intractably ceases to evoke affective processes, even though it appears in every other respect unchanged. The mind makes the inference that the entity that sustains affect, the self, is no longer present. It is important to the account that loss of affect alone is insufficient to produce loss of a sense of self. It is loss of predicted affect (p. 403).

In Gerrans’s model, hypoactivity in the anterior insular cortex (AIC), which is specialized for attributing emotional significance to one’s body state, prevents individuals with DPD from attributing a predicted sense of “mine-ness” to their experience. This hypoactivity is conjectured to result from inhibition by the ventrolateral prefrontal cortex (VLPFC), which plays a general role in the down-regulation of affect. The accounts of Billon and Gerrans each recognize a potential adaptive role for the dissociation of “mine-ness” from self-experience in situations of distressing affect, yet the psychodynamic model has received scant attention in the considerable literature on depersonalization (Sierra and Berrios, 1997).

Building on Gerrans’s model as well as other active inference models (Seth et al., 2012), Deane et al. (2020) view depersonalization as the result of a “loss of allostatic control.” In this model, depersonalization represents the loss of one’s sense of agency from an inability to alter environmental circumstances associated with sustained negative affect.

All of these accounts translate readily into psychodynamic terms. The down-regulation of affect which presumably involves the AIC (Gerrans)may be seen as a defensive operation designed to protect the self from unacceptable or overwhelming affect associated with traumatic or feared experience. Disavowing “mineness” (Billon) as a means of segregating thoughts and feelings is referred to in psychodynamic terms as dissociation. The allostatic control model (Deane et al., 2020) relates depersonalization to a common psychodynamic theme, a fear of losing control.

Where early psychoanalytic models attempted to find confirmation of various psychosexual developmental and energic theories in depersonalization, a less dogmatic approach recognizing the need for grounding in affected individuals may have clinical implications for a psychodynamic or relational approach to depersonalization. In this model, a psychotherapist would not only recognize the clinical importance of appropriate touch and self-touch, but also the symbolic equivalence between touching and knowing that arises in moments of meeting when two wandering minds intersect, or touch each other, in a new way (Lyons-Ruth et al., 1998; Stern et al., 1998; Tronick et al., 1998; Lyons-Ruth, 1999; Fischman, 2022).

### Cognitive loops

The position of *The Thinker* may lend itself to Clark’s (2008, 2017) model of the extended mind, which argues, in effect, that body parts can be viewed as extensions of mind. Clark cites numerous examples of how cognitive processing can be aided by “loops” which include devices outside the brain. For example, the cognitive processing of emotional content can be slowed by Botox injections, which prevent facial muscles from involuntarily contracting while reading the sentence: “Your closest friend has just been hospitalized” (Havas et al., 2010). Alternatively, gel-induced enhancement of facial muscle contraction can increase cognitive processing speed (Neal and Chartrand, 2011).

Clark cites other examples, including using gestures while talking and using fingers while counting, which demonstrate how certain actions can be part of the emotional processing solution. Clark concludes:

At a minimum, such results suggest that the processing of emotion language involves causal-functional loops that run through our own involuntary facial expressions. Loops running through the actual production of these facial expressions (not merely the issuing of neural commands that would normally result in those expressions) thus look to be both functional and integral to the normal processing of such stimuli (2017, p. 7).

It is only a short leap from such reasoning to imagine that pressing one’s fingers or knuckles against one’s lips might facilitate emotional processing in some individuals.

### Affective touch

Ciaunica et al. (2021) propose that “proximal and tactile perceptual engagements with the physical and social world may form a pervasive yet transparent experiential bridge, typically unnoticed and taken for granted. Keeping this “bridge” open is essential in constituting the feeling of being real, present, and immersed in the world.” Ciaunica et al. (2021, 2023) and others who study depersonalization believe that therapies directed at tactile experiencing may help people with depersonalization restore a sense of presence, “reconnect” with their bodies. Much of this hope is based on the discovery and study of CT afferent fibers, which carry emotionally-valenced information referred to as “affectionate touch.”

Fonagy and Campbell (2017) hold that affective (soothing) touch transmits information that the infant decodes regarding the caregiver’s dispositional state and what that state reflects about the infant’s environmental context. In this view, affective touch enables metacommunication of meanings critical to appraising risk and trust. “Through the meeting of physical needs, touch affirms the reality and validity of the infant’s body’s needs.” Would self-touch during states of absorption help re-evoke this sense of the reality and validity of one’s needs, or make the perceived needs of another feel real?

In considering possible motives for self-touch during empathic immersion or other experiences of “losing oneself,” it is intriguing that slow (CT optimal) touch stimulation has been associated with an enhanced sense of body ownership. This association is derived from experiments with the rubber hand illusion (Crucianelli et al., 2013; van Stralen et al., 2014). These results suggest a possible link between the sense of “mine-ness” and CT afferent stimulation. Using an “enfacement illusion,” Panagiotopoulou et al. (2017) provide “direct evidence that embodied affective interactions and particularly affective touch during multisensory integration enhances subjective self-face recognition.” This is an illustration an extra-mind loop in which peripheral CT afferent stimulation facilitates perceptual recognition of one’s self (face). Is it far-fetched to imagine that self-touch, if not through the CT afferent system (a seminal but relatively recent discovery), then through some similar form of tactile-based stimulation, affords some sense of grounding or stamping of experience with “mine-ness” when is immersed in imaginative experience?^6^ The Lennon-McCartney line (“Somebody spoke and I went in to a dream”) reminds us that only the “flimsiest of screens” (James, 1902/2003) separates our rational minds from the surreality of our dreams and other forms of consciousness. As one toggles between the known and the unknown, the pressure of fingers against mouth affords an experiential background, a kind of predictive tactile template that aids in appraising the emotional relevance of newly imagined narratives.

### Philosophical and phenomenological perspectives

There is a long tradition of philosophical ideas regarding the phenomenological experience of touch (for reviews, see Ratcliffe, 2013; Fulkerson, 2020). Jonas views tactile sensation as the linchpin of imagination, a notion that sits well with Winnicott’s observation that the infant’s tactile experiencing becomes linked to “thinking, or fantasying” about the transitional object. “There is a mental side to the highest performance of the tactile sense, or rather, to the use which is made of its information, that transcends all mere sentience, and it is this mental use which brings touch within the dimension of the achievements of sight. It is the image-faculty, in classical terms: *imaginatio*, *phantasia*, which makes that use of the data of touch” (Jonas, 1954, p. 511). According to Jonas, of all the senses it is touch which brings a sense of reality to perception because it incorporates reciprocal force, rendering the subject of experience active in perceiving the object, not merely passive.

Ratcliffe, following Jonas, argues that the sense of “touch is partly constitutive of the sense of reality and belonging, whereas other kinds of sensory experience presuppose it. Hence, touch has a kind of phenomenological primacy over the other senses” (2013, p. 132). This philosophical appraisal of the primacy of the sense of touch in relation to the other four primary senses extends back to Aristotle. Bertrand Russell’s view is typical of these contributions: “It is touch that gives us our sense of ‘reality’.… Our whole conception of what exists outside us, is based upon the sense of touch” (Russell, 1925, p. 10).

Touch is unique among the senses in other ways, too. As summarized by Ratcliffe (2013), the phenomenologists Husserl and Merleau-Ponty both recognize the unique place of touch in the act of touching oneself, in which one is simultaneously the one touching and the one being touched. However, phenomenologically, one does not experience oneself as subject and object at the same time; it is reciprocally one, then the other. For Merleau-Ponty, this duality of touching and being touched, or perceiving and being perceived, is the model for intersubjectivity. In touching oneself, experiencing oneself as an object, one forms an idea that one exists as an object for others. The reciprocity between being subject and object of touch when one touches one’s own hand is extended when one touches and is touched by the hand of another. In this case, one feels that one is in contact with another subject for whom one is an object. Here *touching and being touched is the paradigm for intersubjective knowing and being known*.

It also suggests a way of understanding one of the meanings or functions of pressing one’s fingers against one’s mouth when pondering the other. In activating the reciprocal relationship between being the subject and object of touch, one activates the reciprocity between being the subject and object of intentions that lies at the core of intersubjective sharing.

Korsmeyer (2012) discusses a dimension of touching that, paradoxically, suggests contact may not be required. She discusses the “transitive” qualities of touch related to our esthetic sense of the genuineness of objects. She considers why the lines outside the Library of Congress in 2009 were blocks long to see a display of Lincoln’s original Gettysburg Address, but there was no line to see an identical facsimile. The answer is that there is something experientially quite different about witnessing something that Lincoln actually touched than something he never laid hands on. Korsmeyer concludes that touch has “transitive” properties that are conveyed across time, even millennia, and across space via an “aura” that one experiences when within certain proximity of a significant object. This supports O'Shaughnessy’s (2000) startling contention that “tactile sensation is inessential to tactile perception” (p. 662). Korsmeyer acknowledges that the thrilling sense of connection one feels to the object’s creator in these experiences of proximity is based upon magical thinking. To defend magical thinking as valid grounds for feelings of genuineness, she resorts to more epistemically acceptable notions such as “primitive intelligibility,” “cognitive penetrability,” and “non-fungible emotions.”

But magical thinking needs no defense as a component of pondering. While our secondary process thinking may override primary process-derived magical thinking much of the time, we are never more than an utterance away from it. Korsmeyer’s recognition that the transitive properties of touch enable imagined communion with idealized objects highlights another way in which touch may facilitate knowing. Proximity to the genuine article incites feelings of awe and wonder through an imagined sense of proximity with the article’s idealized creator. The magic in this may derive from evoking feelings associated with attachment to the omnipotent caregiver of infancy/early childhood. If mere proximity can incite a subjective experience of contact, it becomes easier to posit that certain forms of self-touch may function imaginatively in the same way.

### Rodin (slight return)

Albert Elsen, the Rodin scholar, quotes a statement Rodin made about creating *The Thinker* in a letter he wrote to the critic Marcel Adam, printed in *Gil Blas* (July 7, 1904).

*The Thinker* has a story. In the days long gone by, I conceived the idea of *The Gates of Hell*. Before the door, seated on a rock, Dante thinking of the plan of his poem. Behind him, Ugolino, Francesca, Paolo, all the characters of *The Divine Comedy*. This project was not realized. Thin, ascetic, Dante in his straight robe separated from the whole would have been without meaning. Guided by my first inspiration I conceived another thinker, a naked man, seated upon a rock, his feet drawn under him, his fist against his teeth, he dreams. The fertile thought slowly elaborates itself within his brain. He is no longer dreamer, he is creator (2003, p. 175).

**Figure 3:**
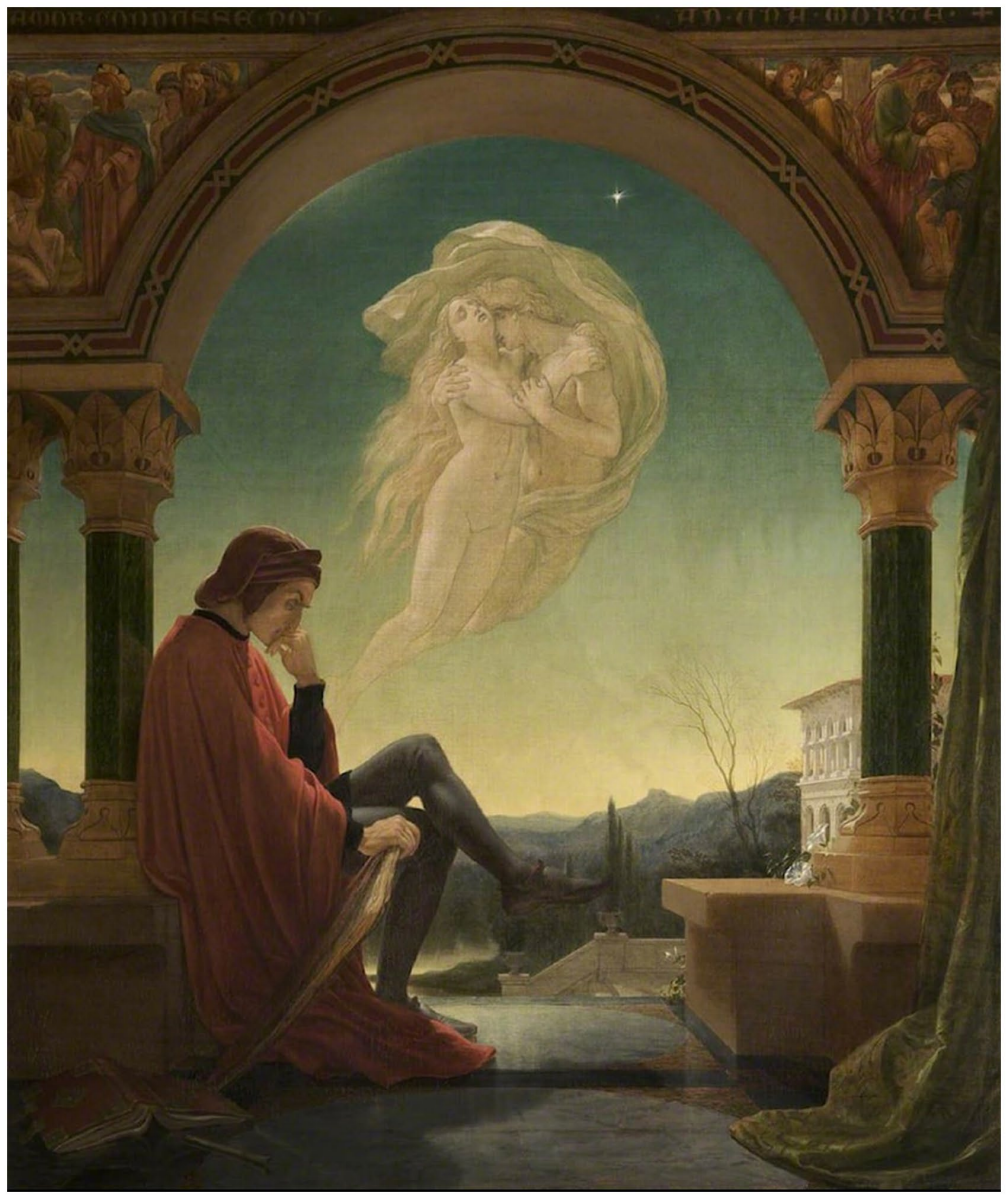
Dante Meditating the Episode of Francesca da Rimini and Paolo Malatesta, by Joseph Noel Paton (1821-1901). Photo credit: Bury Art Museum. Reproduced with permission.

For Rodin, the key difference is that in the first concept, the subject is in a straight robe and “separated” from the characters in his mind (Figure 3; compare with Figure 1), while in the second, the subject is not “separated from the whole.” His body is in multiple points of tactile contact with himself and his surround. He is naked. He is not “thin, ascetic,” but thickly muscular, an anatomical record of tactile exertion. He is seated firmly upon a rock, not just with haunches on the edge of a seat, but with legs drawn up under his torso, feet firmly anchored to the stone beneath them; right fist mashed against teeth; right elbow burrowed into thigh muscle; left arm and hand draped over left knee (Figure 1). And with all this tactile grounding, he “dreams;” not idle dreaming, but ‘creating.’ That is, he is slowly elaborating new, original thoughts about the objects he is pondering, just as Rodin finally did as he contemplated his subject, Dante, the poet, planning his poem.

**Figure 4:**
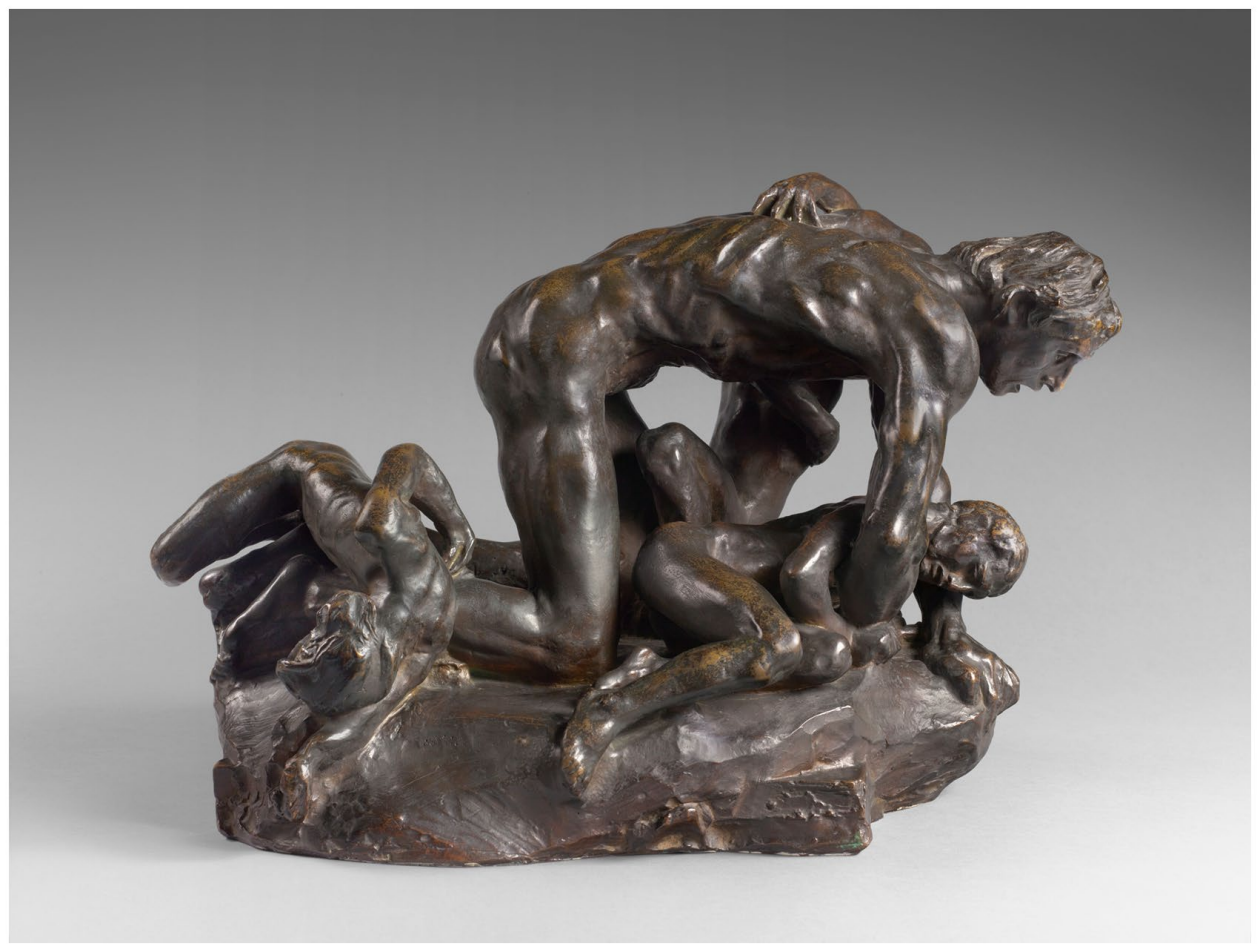
Ugolino and his Children by Auguste Rodin, S.1146 (41 cm x 61,5 cm x 41 cm) © Musée Rodin – photo Hervé Lewandowski. Reproduced with permission.

We recall (see above) Rodin’s 1890 statement about how he lived a whole year with Dante alone drawing the circles of Dante’s *Inferno*, only to feel that he, or at least his drawing, had grown too far removed from reality. Along the way, Rodin, who described Dante’s poetry as sculptural (Elsen, p. 157), identified with Dante’s creative process to such an extent that he needed to distance himself from Dante to create something original. With his 1904 statement, and in the figure of *The Thinker* itself, we can appreciate more fully his artistic rendering of his felt need to leave Dante’s vision and to ground his creative process by working directly again with models. Nothing illustrates this process of grounding more clearly than Rodin’s ultimate decision to situate *The Thinker* over his own grave, creating in death a lasting image of being grounded while absorbed in thought.

### Rodin, Dante, Ugolino, and ambiguity

By making *The Thinker* his headstone, Rodin also memorialized his position next to Dante, from whom he once needed to distance himself. This observation seems an apt way to introduce the role of ambiguity in linking touching with “thinking, or fantasying.” In a study of ambiguity as a means of eliciting and refining self-other discrimination, de Bézenac et al. (2018) quote Ramachandran and Rogers-Ramachandran (2008): “The brain abhors ambiguity, yet we are curiously attracted to it.”

To avoid lengthy digression, the reader unfamiliar with the Ugolino story is referred elsewhere (the Wikipedia entry “Ugolino della Gherardesca” is a good start). Here, it must suffice to say that Dante creates ambiguity deliberately by the way he narrates the tale: having the shade, Ugolino, tell his version of his story to Dante, the pilgrim. The reader is left to sort out whether Ugolino’s horrible fate is a just punishment for his traitorous act; whether the pilgrim should pity Ugolino and decry Ruggieri’s punishment, which includes starving Ugolino’s innocent children; and particularly, whether Ugolino’s famous line, “Famine did what starvation could not” (Canto 33, line 75) implies that he cannibalized his children.

Elsen (p. 205) states that Rodin became practically obsessed with Dante’s Ugolino. Ramachandran and Rogers-Ramachandran’s statement about ambiguity may explain why. Rodin went through various stages in his decision about how to depict this scene (Elsen, p. 200), ultimately molding Ugolino on his hands and knees on top of the bodies of his dead and dying children, groping blindly, cradling a dying child with one arm as another child loses his weak grip on Ugolino’s back (Figure 4). I suspect Rodin ultimately chose and created this scene because when Ugolino can no longer see, he feels his way through the horror of his fate, literally feels his children dying against his body. It is only through tactile contact with their dying bodies that he finally comes to know the cruel irony of his punishment. This scene of Ugolino on all fours evokes Ugolino’s dream of himself and his children as hunted wolves, a dream from which Ugolino awakens to hear his children crying in their sleep from hunger. Rodin’s emphasis on the tactile elements of the scene reinforces Dante’s notion of truth being revealed by feelings from within, not by their appearance.

All of this is consistent with de Bézenac et al.’s (2018) theories about ambiguity. The reality testing mechanisms called into play in resolving ambiguity are the same ones called into play early in life in distinguishing self from other. These include recognizing invariant qualities which cohere together and are therefore likely to belong to a single entity, and variable qualities which do not cohere and are therefore unlikely to belong to a single entity. Invariants include the sense of agency, which derives from perceiving little to no difference between actual and predicted consequences of actions. These models of self and other can be challenged by circumstances in which coherence is a property of multiple entities acting in concert, and when incoherence is a property of a single entity. This is how ambiguity may arise.

De Bézenac et al. (2018) state that:

Ambiguity (as fundamental characteristic of many everyday social encounters) plays a key role in developing the sense of self and in learning to differentiate between the boundaries of objects including those that exist between self and other as agents in the world. We propose that engagement in challenging activities that require self–other differentiation may provide optimal conditions for refining reality-testing abilities related to self–other processing.

Such engagement includes “absorbed participation in music and arts,” which “enriches perception through the re-encountering of self in novel ways.” De Bezenac et al. suggest that creativity emerges from “an increasingly refined awareness of, and playful engagement with the boundaries between internal and external experience – through a fluid interplay between the two.” In other words, creativity is enhanced by deliberately engaging ambiguity.

Ambiguity may be why Dante included the stories of Francesca and Paolo, and Ugolino in *The Inferno*, and why Rodin became practically obsessed by Dante’s Ugolino. Ambiguity engages the same strategy (predictive processing) organisms employ to regulate homeostasis to insure survival. “Expected free energy can be decomposed into *risk* and *ambiguity*…. (Therefore) minimizing ambiguity becomes a fundamental (existential) imperative” (Friston, 2020, p. 58). In the case of vicarious absorption in a wrenching narrative about the life and death of fellow human, we feel compelled to reckon with how we would handle similar circumstances. De Bezenac et al. conclude that promoting play in the “in-between space” [i.e., Winnicott’s (1971a,b) transitional space] during childhood, and absorption in ambiguity fostered by music and the arts in adulthood lead to greater resiliency, flexibility, and adaptability to stressful environments.

Thus, ambiguity affords space and time to ponder, to immerse oneself empathically in the quandary of another and through imaginative trials test ways of dealing with adversity. These trial actions are appraised by how they make one feel. After years of pondering Dante’s Ugolino, Rodin concluded that only through touch could Ugolino know the horror of his punishment, feel the suffering he brought to his children. To paraphrase Dante’s famous line in this tale, “Touch did what seeing and hearing could not.”

## Conclusion

It is through the background sensation of touching and being touched, of alternately being both the subject and object of experience (Merleau-Ponty, 1962; Husserl, 1989; Ratcliffe, 2013) that cognition or trial action with regard to another’s imagined experience is joined with “me” feelings. In his preparation for a talk on *The Fate of the Transitional Object* (1989), Winnicott explicitly discusses how the infant’s exploratory caressing lends a sense of reality to experience. “In this way, we can see that the infant’s use of an object can be in one way or another joined up with body functioning, and indeed, one cannot imagine that an object can have meaning for an infant unless it is so joined. This is another way of stating that the ego is based on a body ego.” One can imagine Winnicott’s infant joining fingers and mouth in transitional space, exploring the sensation of being both subject and object of touch.

Montagu emphasizes the outsized representations of the fingers and lips in both the sensory and motor human homunculi. He writes,

It is through the lips that the infant grasps reality, as well as the body-building substances that he ingests. It is for a time the only means of judgment the infant has. That is why, as soon as he is able, he puts things to his lips in order to judge them, and continues to do so long after he has arrived at other means of perception and judgment. The other means of perception and judgment at which the infant ultimately arrives are through the tips of his fingers and the palms of the hand…. While all his senses are operative and play an increasingly significant role in his perception and communication with the external world, especially with the mother, none are as basic as touch. It is the sense of touch upon which the infant depends: lips, and generalized body contact, and then fingertips to whole hand (1971, p. 129).

Few would doubt that the fingers and lips play a crucial role in judging reality early on in development. Ultimately, I only suggest that they may continue to play this role in later life, as an extension of mind (Clark, 2008, 2017) when one loses oneself, so to speak, in an absorbing narrative. In such absorption, self-touch affords tactile grounding, a message of mine-ness while sampling otherness. The phenomenon of depersonalization shows how easily ipseity can be lost. Self-touch helps translate vicarious experience into familiar (predicted) haptic substrates, reminiscent of our first encounters in the transitional space between subjective reality and that which is objectively perceived.

Our models of the brain are subject to fairly rapid change. The models of depersonalization discussed above suggest that attenuation of somatosensory information generated through action is crucial in maintaining transparency, or a sense of presence with regard to perception, as well as a sense of agency (Ciaunica et al., 2021). Situations of ambiguity or complex emotional significance are perceived as a threat to self-preservation, which increases the precision (attention) of self-monitoring functions, exactly the opposite of what is needed for transparency during empathic immersion. Optimal precision-weighting requires flexible, context-sensitive adaptation (Ciaunica et al., 2022). Self-touch supplies a predicted but sufficiently attenuated sense of self just when self-representation must be suppressed or switched off (Bird and Viding, 2014; Haggard, 2017). It is possible that self-touch optimizes precision-weighting between prior expectation and sensory input. Familiar comforting pressure on the lips, for example, might circumvent potential hyperreflexivity or loss of control in relation to perceived threat by enabling a predicted transparent “experience of being in immediate relation to a self” (Limanowski and Friston, 2018), or what one subjectively experiences as agency or “grounding” as one approaches the foreboding shades of one’s imagination.

Behavioral and functional neuroimaging correlation studies in this area are just beginning. Intriguingly, positive correlations were found between spontaneous facial self-touch (sFST) and increases in pre-frontal theta and alpha power when subjects were distracted while absorbed in a complex retention task (Grunwald et al., 2014; Spille et al., 2022a), while suppression of sFST impaired memory performance for “high” sFST subjects (Spille et al., 2022b).

As Ratcliffe puts it, “Central to our sense of reality is the experience of affecting and at the same time being affected by things. Vision offers us ‘a calmed abstract of reality denuded of its raw power,’ whereas touch supplies what it lacks and is therefore the ‘true test of reality’” (2013, p. 133). It is through this simultaneous experience of affecting and being affected by things that self-touch grounds the subject who is “thinking, or fantasying” of others in a sense of reality.

As suggested above, anxiety associated with losing one’s sense of agency may interfere with mind-wandering (Hohwy’s seesaw) between self-reflection and absorptive (self-less) states in depersonalization. That fluidity, and the grounding function of self-touch in pondering, are best conveyed experientially. “A Day in the Life” conveys this transition eloquently in music and in lyrics. Dante achieves this by structuring his poem around an alter-ego, a pilgrim, who travels through *The Inferno* guided by a poet and contacts directly the shades of the underworld. Dante may have inserted the conceit of Francesca and Paolo reading Galehaut’s story (of Guinivere and Lancelot) as a way of illustrating that without alternately grounding oneself in self-reflection, vicarious immersion may carry one too far from one’s ideals and values. This was the lesson learned by Rodin after immersing himself for a year in Dante’s world. Self-touch grounds experience within one’s sense of reality. Rodin’s *The Thinker* embodies that grounding.

## Data availability statement

The original contributions presented in the study are included in the article/supplementary material, further inquiries can be directed to the corresponding author.

## Author contributions

The author confirms being the sole contributor of this work and has approved it for publication.

## Conflict of interest

LF is an independent contractor for Fluence. The company was not involved in the study design, collection, analysis, interpretation of data, the writing of this article or the decision to submit it for publication.

## Publisher’s note

All claims expressed in this article are solely those of the authors and do not necessarily represent those of their affiliated organizations, or those of the publisher, the editors and the reviewers. Any product that may be evaluated in this article, or claim that may be made by its manufacturer, is not guaranteed or endorsed by the publisher.

## Footnotes

^1^Allen Mandelbaum translation.

^2^HF Cary translation.

^3^In their valuable tome on psychedelic experience, Masters and Houston (1966, p. 130) cite the character of Virgil in Dante’s *Inferno* as a model for the psychedelic psychotherapist, when Virgil says to the pilgrim (Dante) who is lost in the dark woods, “I shall be your guide to lead you hence through that eternal place” (Dante’s *Inferno*, Canto 1, lines 113–114).

^4^Curiously, Browne’s girlfriend, who was in the car with Browne at the time of the crash, but escaped injury, insisted that Browne swerved to avoid a car that would have slammed into her. In a well-known version of the Francesca and Paolo story, Giovanni intended to stab his offending brother when Francesca threw herself in front of Paolo and took her husband Giovanni’s sword’s thrust herself.

^5^Hohwy has a separate schema for the “minimal” self. It is pre-reflective, related to a sense of agency, and to a sense of “mine-ness,” which is given by “the experience of having predicted, and thus already being familiar with, what one perceives” (p. 8; see also Zahavi and Parnas, 1998; Gallagher, 2000; Metzinger, 2003).

^6^Fulkerson (2020) asks, “Are there other similar receptors for pleasant touch in glabrous skin (the smooth, hairless skin of the lips and palms) where there are no CT–afferents?”
